# A novel dual-drainage system for managing a duodenal stump leak with intra-abdominal hemorrhage postgastrectomy: A case report

**DOI:** 10.1097/MD.0000000000046796

**Published:** 2026-03-13

**Authors:** ZhuoDong Li, Wei Chai, Xin Yang, YingRou Liu

**Affiliations:** a Cangzhou Central Hospital of Hebei Medical University, Cangzhou, Hebei, China; b Department of Hepatobiliary and Pancreatic Surgery, Cangzhou Central Hospital of Hebei Medical University, Cangzhou, Hebei, China.

**Keywords:** case report, drainage, duodenal stump leak, gastrectomy, intra-abdominal hemorrhage, irrigation, T-tube

## Abstract

**Rationale:**

Duodenal stump leakage followed by intra-abdominal hemorrhage is a rare but serious complication of gastrectomy. Managing this condition is highly challenging. We describe a novel approach using a combined intra- and extraluminal drainage system that proved to be a simple, safe, and effective solution.

**Patient concerns:**

A 66-year-old male with a history of hypertension and coronary artery disease presented with a 1-year history of epigastric distension and discomfort. After undergoing radical total gastrectomy for gastric adenocarcinoma, he developed increased abdominal drain output and pain, followed by acute hemorrhage from the drain site.

**Diagnoses:**

Postoperative duodenal stump leak (diagnosed on postoperative day [POD] 6 by computed tomography scan) complicated by subsequent rupture and hemorrhage of the left hepatic artery (confirmed on POD 10 during emergency laparoscopy).

**Interventions:**

The initial duodenal leak (POD 6) was managed with computed tomography-guided percutaneous catheter drainage. On POD 10, due to an acute intra-abdominal hemorrhage, emergency surgery was performed. After securing hemostasis of the left hepatic artery and primary suturing of the duodenal leak, a 20-French T-tube was inserted into the duodenum for intraluminal decompression. A double-cannula system was then placed adjacent to the leak site for continuous extraluminal irrigation and suction.

**Outcomes:**

Following the procedure, the patient’s condition improved steadily. The double-cannula irrigation system was removed on POD 30, and the patient was discharged. The T-tube was clamped on POD 30 and successfully removed on POD 48. The patient recovered completely without further complications.

**Lessons:**

The technique of T-tube intraluminal drainage combined with continuous extraluminal irrigation via a double-cannula system provides an effective management strategy for duodenal stump leakage complicated by intra-abdominal hemorrhage. This case highlights that optimal outcomes in such complex scenarios are best achieved through a comprehensive strategy that integrates innovative surgical techniques with systematic perioperative nutritional and metabolic support.

**Key Points**

1. The combination of intraluminal drainage and extraluminal irrigation represents a simple, safe, and effective treatment approach.
2. Duodenal stump leakage complicated by intra-abdominal hemorrhage is a rare and life-threatening complication.
3. Our intraoperative management of arterial hemorrhage and duodenal stump leakage achieved remarkable therapeutic efficacy.
4. Maintaining a clean wound bed and ensuring patency of drainage are critical for successful treatment and improved patient prognosis.

## 1. Introduction

Duodenal stump leakage (DSL) is an uncommon yet serious complication following distal or total gastrectomy.^[1]^ The leakage of corrosive duodenal contents into the abdominal cavity can lead to severe sequelae, including complex intra-abdominal infections, sepsis, and hemorrhage, which may become life-threatening.^[2–4]^ Conventional management often involves drainage placement near the leak site, with T-tube duodenostomy representing an established technique for DSL.^[1,4]^ This report describes a case where DSL resulted in rupture and hemorrhage of the left hepatic artery. To address both the leakage and prevent recurrent bleeding, we implemented a technique employing T-tube intraluminal drainage through the duodenal bulb-descending junction combined with continuous extraluminal irrigation and drainage via a double-cannula system. This study aims to detail the technical aspects and clinical outcomes associated with this approach.

## 2. Case presentation

A 66-year-old Asian male with a medical history of hypertension and coronary artery disease presented with a 1-year history of epigastric distension and discomfort. Esophagogastroduodenoscopy revealed a mass extending from the subcardiac region to the anterior gastric wall. Endoscopic biopsy confirmed the diagnosis of poorly differentiated tubular adenocarcinoma. The patient subsequently underwent radical total gastrectomy with Roux-en-Y esophagojejunostomy reconstruction.

On postoperative day (POD) 6, the abdominal drain output increased slightly, yielding clear, pale yellow fluid. The patient experienced mild abdominal distension. Apart from a mildly elevated white blood cell count (11.6 × 10^9^/L), his vital signs and other laboratory parameters remained stable. An emergency computed tomography (CT) scan of the abdomen and pelvis revealed a small, localized fluid collection with minimal air near the duodenal stump, consistent with DSL. Consequently, CT-guided percutaneous catheter drainage was performed, with the catheter draining ~200 mL of bile daily.

**Figure 1.**
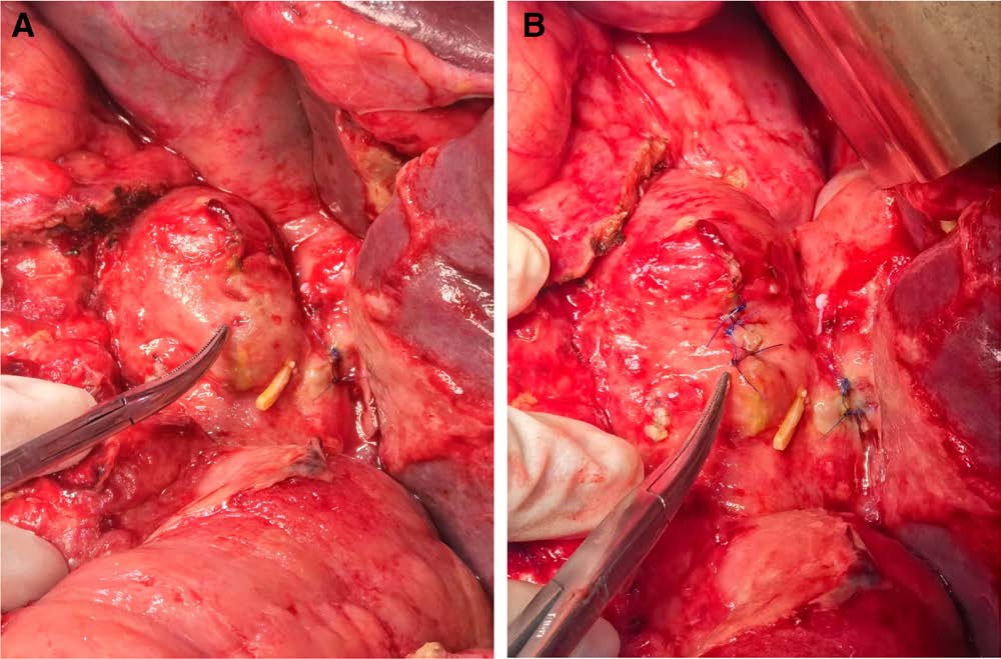
(A) Identification of a small defect at the duodenal stump. (B) Primary suturing of the identified leak.

On POD 10, the patient experienced acute abdominal pain accompanied by bleeding from the drainage site, necessitating urgent diagnostic laparoscopy. Intraoperative findings included 500 mL of hemoperitoneum. After lysing adhesions and debriding the abscess cavity surrounding the duodenal stump, active bleeding was identified from the anterior wall of the left hepatic artery. Following hemostasis, further exploration revealed a small defect at the duodenal stump with extravasation of enteric contents and significant tissue edema (Fig. 1A). The leak was managed with primary suturing (Fig. 1B).

**Figure 2.**
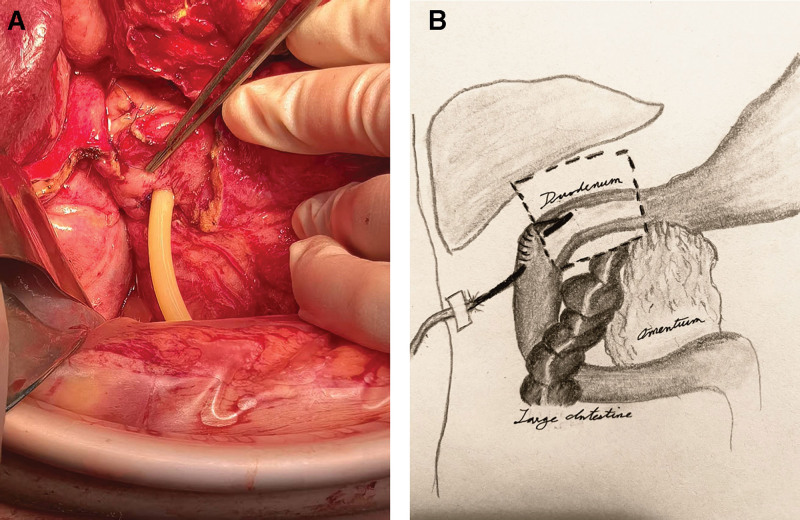
(A) T-tube placement through the duodenal bulb-descending junction for intraluminal drainage. (B) Illustration of T-tube duodenostomy fixed via the Witzel technique (illustration by Emma Rousakis, MD).

**Figure 3.**
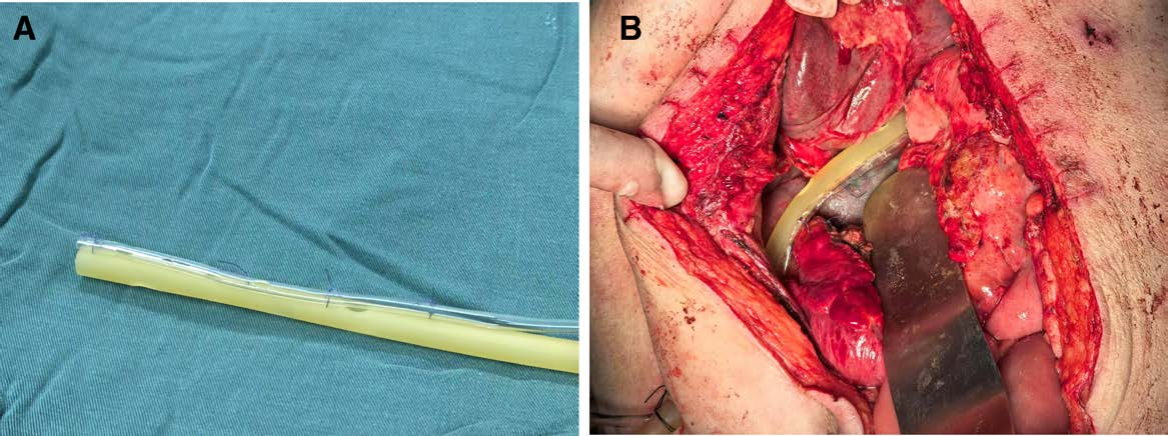
(A) Construction of the double-cannula system. (B) Placement of the double-cannula system for extraluminal irrigation and drainage.

Given the high probability of DSL recurrence, a 20-French T-tube was inserted through the lateral duodenal wall at the bulb-descending junction. The T-tube was inserted into the intestinal lumen (Fig. 2A) and then secured in place using the Witzel technique to establish intraluminal drainage (Fig. 2B). Subsequently, a double-cannula system was created by suturing an abdominal drainage tube parallel to an infusion tube (Fig. 3A). This system was positioned near the leak site for continuous extraluminal irrigation and drainage (Fig. 3B). Intraoperatively, the patient received 4 units of packed red blood cells and 4 units of fresh frozen plasma.

Postoperatively, outputs from the T-tube and irrigation system were meticulously monitored. From POD 1 to 21, T-tube output fluctuated between 60 and 550 mL, while irrigation input and output volumes remained balanced. By POD 28, T-tube output had decreased to <30 mL per day. A follow-up CT scan showed no abnormalities. On POD 30, the T-tube was clamped without discomfort, the double-cannula system was removed, and the patient was discharged. The T-tube was successfully removed on POD 48, and the patient recovered well thereafter. This modified dual-drainage technique offers a novel strategic approach for managing DSL complicated by intra-abdominal hemorrhage following gastrectomy.

## 3. Discussion

DSL is a serious complication that can arise from various etiologies, including resection, injury, or repair of perforation.^[1,2]^ In recent years, its incidence has decreased to 1.1% to 2.5%, with mortality rates ranging from 15% to 75%.^[5]^ DSL typically manifests between 6.6 and 9 days postgastrectomy.^[5–7]^ The leakage of corrosive duodenal fluid into the abdominal cavity can trigger severe complications, including complex infections, sepsis, and hemorrhage.^[6,7]^ Surgical intervention in the context of tissue inflammation and friability increases the risk of re-leakage. Furthermore, the duodenal stump cannot be exteriorized to divert corrosive fluid. In such scenarios, intraluminal drainage tube placement in the duodenal bulb-descending region can effectively decompress the duodenum, reduce pressure on the stump, and divert leakage externally, thereby transforming DSL into a controllable enterocutaneous fistula.

In this case, although hemostasis of the left hepatic artery was achieved, a double-cannula system was placed near the leak site to prevent early leakage of duodenal fluid that could potentially corrode the vascular wall. One tube was used for continuous irrigation with normal saline, while the other was connected to a drainage bag to dilute corrosive fluid and reduce the risk of rebleeding and intra-abdominal infection. The advantages of this technique include Effective diversion of duodenal fluid, reducing stimulation at the leak site; continuous irrigation and drainage, maintaining a clean peri-leak environment; reduced risk of abdominal infection and arterial rebleeding; and creation of favorable conditions for leak healing. In this particular case, the treatment achieved excellent therapeutic outcomes.

The successful management of this case underscores not only the value of innovative surgical techniques in addressing local complications but also highlights the critical necessity of integrating systematic nutritional support into the perioperative care of patients undergoing complex surgeries. Recent advancements in the field of oncology nutrition increasingly emphasize the importance of a multidisciplinary approach. A recent national survey indicates that while awareness and establishment of clinical nutrition programs among Italian oncologists have significantly improved (with 80.8% of respondents reporting such programs in their institutions), the representation of dedicated nutrition specialists within multidisciplinary teams remains suboptimal (at only 26.0%), which may hinder the optimal implementation of nutritional interventions.^[8]^ This finding resonates strongly with our experience. For this patient in a hypermetabolic state complicated by DSL and intra-abdominal hemorrhage, alongside the local control achieved through T-tube drainage combined with double-cannula irrigation, we implemented an early, personalized nutritional support protocol strictly adhering to ESPEN/ERAS guidelines. This protocol included high-energy, high-protein oral nutritional supplements and immunonutrition enriched with arginine and omega-3 fatty acids. The comprehensive strategy, which equally prioritized “surgical technical intervention” and “systemic metabolic-nutritional support,” likely played a pivotal role in creating a favorable internal milieu for tissue repair by mitigating systemic inflammation and correcting negative nitrogen balance, thereby forming the cornerstone of the patient’s successful recovery. Consequently, the experience from this case reinforces the notion that the optimal management strategy for patients suffering from severe complications after gastrectomy should transcend a purely technical solution. Instead, it is imperative to embed structured, guideline-recommended nutritional care as an integral component throughout the entire treatment course.

## 4. Conclusion

The technique of T-tube intraluminal drainage combined with continuous extraluminal irrigation via a double-cannula system represents a simple, safe, and effective therapeutic approach for the challenging scenario of DSL complicated by intra-abdominal hemorrhage. Crucially, this case demonstrates that the success of such technical interventions is significantly enhanced when they are embedded within a comprehensive management strategy. This strategy integrates targeted surgical innovation with rigorous, guideline-adherent nutritional and metabolic support, addressing both the local complication and the systemic catabolic state. We propose that this combined technical and systemic approach offers a superior paradigm for managing complex surgical complications. Future efforts should focus not only on standardizing the procedure but also on ensuring the consistent application of multidisciplinary care principles, including perioperative nutrition, to optimize patient outcomes across diverse clinical settings.

## Author contributions

**Conceptualization:** Wei Chai.

**Data curation:** ZhuoDong Li.

**Investigation:** Xin Yang.

**Methodology:** Wei Chai.

**Project administration:** ZhuoDong Li, Wei Chai.

**Supervision:** YingRou Liu.

**Writing – original draft:** ZhuoDong Li.
